# Tailoring and Remotely Switching Performance of Ultrafiltration Membranes by Magnetically Responsive Polymer Chains

**DOI:** 10.3390/membranes10090219

**Published:** 2020-09-01

**Authors:** Anh Vu, Arijit Sengupta, Emily Freeman, Xianghong Qian, Mathias Ulbricht, S. Ranil Wickramasinghe

**Affiliations:** 1 Ralph E Martin Department of Chemical Engineering, University of Arkansas, Fayetteville, AR 72701, USA; anhtvu1985@gmail.com (A.V.); arijitbarc@gmail.com (A.S.); freemae@email.sc.edu (E.F.); 2 Bhabha Atomic Research Centre, Mumbai 400085, India; 3 Department of Biomedical Engineering, University of Arkansas, Fayetteville, AR 72701, USA; xqian@uark.edu; 4 Lehrstuhl für Technische Chemie II, Universität Duisburg-Essen, 45117 Essen, Germany; mathias.ulbricht@uni-essen.de

**Keywords:** atom transfer radical polymerization, concentration polarization, flux, fouling, rejection

## Abstract

Magnetically responsive ultrafiltration membranes were prepared by grafting poly(2-hydroxyethyl methacrylate) chains from the outer surface of 100-kDa regenerated cellulose ultrafiltration membranes. Surface-initiated atom transfer radical polymerization was used to graft the polymer chains. Grafting from the internal pore surface was suppressed by using glycerol as a pore-filling solvent during initiator immobilization at varied densities. Glycerol suppresses the initiator attachment to the pore surface. Polymerization times of up to four hours were investigated. Superparamagnetic nanoparticles were covalently attached to the chain end. Membrane performance was determined using bovine serum albumin and dextran as model solutes. Increasing the grafted polymer chain density and length led to a decrease in the permeate flux and an increase in the apparent rejection coefficient. In an oscillating magnetic field, movement of the grafted polymer chains led to a decrease in the permeate flux, as well as an increase in the apparent rejection coefficient of the model solutes.

## 1. Introduction

In recent years, the ability to develop membranes that respond to an external stimulus has attracted a great deal of attention [1,2,3]. These membranes offer the possibility of tuning membrane performances based on the prevailing environmental conditions. Numerous external stimuli have been investigated, such as pH, ionic strength, temperature, electromagnetic radiation and electrical and magnetic fields. This contribution focuses on the development of magnetically responsive ultrafiltration membranes.

Magnetic nanoparticles can be trapped within the membrane matrix, attached to the surface of the membrane or tethered via a polymer chain from the membrane surface. Further, in the case of porous ultrafiltration membranes, they can be entrapped in a hydrogel within the membrane pores [4,5]. In the presence of a magnetic field, magnetic nanoparticles that are trapped within the membrane matrix will experience a force and a torque that can lead to magnetophoretic actuation and alignment. In addition, in an oscillating magnetic field, magnetic nanoparticles can induce heating.

In ultrafiltration, mass transfer is by convective flow to the membrane surface and through the membrane pores. Several researchers have investigated the possibility of inducing localized heating when magnetic nanoparticles are subjected to an oscillating magnetic field. In a recent study, Tang et al. developed magnetically responsive mesoporous block copolymer membranes [6]. Magnetic nanoparticles were embedded in the mesoporous selective layer of a composite membrane containing a macroporous PVDF support structure. The selective layer consisted of poly(oligo(ethylene glycol) methyl ether methacrylate)-block-polystyrene-block-poly(oligo(ethylene glycol) methyl ether methacrylate), which is a thermo-responsive polymer. In an oscillating magnetic field, heating induced by the magnetic nanoparticles led to an increase in the temperature of the selective layer above its LCST, leading to a change in the effective pore size. Thus, the membrane performance could be tuned.

Lin et al. used a different approach [7]. They developed a mixed matrix composite membrane based on polyether sulfone as the membrane material blended with PNIPAm hydrogel microparticles and magnetic iron oxide nanoparticles, prepared by one-step film casting and phase inversion. In the presence of an oscillating magnetic field, heating induced by the nanoparticles led to the temperature of the barrier layer increasing above its LCST and deswelling of the PNIPAm particles, thus opening additional pathways through the barrier layer. This led to an increase in water flux and nominal molecular weight cutoff of the membrane.

Ng et al. used another approach. Polyelectrolyte multilayers were deposited on the surface of a polyether sulfone membrane [8,9]. Magnetic nanoparticles were trapped in the polyelectrolyte multilayer. Magnetophoretic actuation reduced fouling by humic acid and improved the membrane performance. Azmi et al. showed that movement of the nanoparticles could lead to a decrease in concentration polarization [10]. Low et al. extended the work by investigating the use of a base cellulose acetate membrane [11].

We have used yet a different approach. Using atom transfer radical polymerization (ATRP), we have grafted polymer chains from the surface of nanofiltration and microfiltration membranes. We have then attached a superparamagnetic nanoparticle to the chain end. In a magnetic field, the particles experience a force and a torque. In addition, in an oscillating magnetic field, the particles can induce heating. We have made use of these effects in a number of applications. When poly(2-hydroxyethyl methacrylate) (PHEMA) is grafted from the surface of nanofiltration membranes, we show that movement of the grafted polymer chains can break up concentration polarization and suppress fouling [12,13,14,15,16]. When these same magnetically responsive polymer chains are grafted from the inside pore surface of track-etched polyethylene terephthalate membranes, changes in the grafted polymer conformation lead to remote-controlled valves where a magnetic field can be used to modulate the membrane performance [17].

Grafting PNIPAm from the surface of nanofiltration and microfiltration membranes and then attaching a superparamagnetic nanoparticle to the chain end allowed us to change the conformation of the grafted polymer chains not only by movement but, also, by heating induced in an oscillating magnetic field [18]. These effects may be used to modulate the permeate flux and rejection of the membrane.

As nanofiltration membranes have very small pores, grafting will occur from the barrier layer. Thus, the effects of changes in the conformation of the grafted polymer chains will be due to polymer chains grafted from the barrier layer. Diffusional limitations combined with steric hindrance will minimize the chain growth from initiator molecules attached to the inner pore surface. Microfiltration membranes, however, represent the opposite extreme. While grafting will occur from the outer membrane surface, as well as the inner pore surface, the effects of the changes in the conformation of grafted polymer chains will be due to polymer chains grafted from the inner pore surface. In the case of ultrafiltration membranes, which have pore sizes between nanofiltration and microfiltration membranes, the effect of the changes in the conformation of the grafted polymer chains will be different to both the microfiltration and nanofiltration membranes.

The modification of ultrafiltration membranes, however, is problematic for two reasons. While ATRP is a controlled polymerization that enables the growth of relatively monodisperse polymer chains, it is not location-specific. Polymer chains will grow from the inside pore surface, as well as the external membrane surface, wherever an initiator molecule has attached. The growth of polymer chains inside the membrane pores will change the membrane rejection properties and can lead to pore plugging. Secondly, ATRP must be conducted in an oxygen-free environment where the reaction solvent, frequently water, must be deoxygenated. Further, the membranes are often dried after various modification steps. This can lead to a collapse of the membrane pore structure. Our recent work overcomes these limitations [19]. We have shown that, by using glycerol as a pore-filling solvent, we can suppress the growth of polymer chains inside the pores. By using activator-generated electron transfer (AGET) ATRP, we avoid the need to deoxygenate the reaction solvent or dry the membrane between modification steps.

Here, we use AGET ATRP to graft PHEMA from the surface of regenerated cellulose ultrafiltration (RCUF) membranes and, subsequently, attach a superparamagnetic particle to the chain end. We have, for the first time, used our surface modification methods to develop magnetically responsive ultrafiltration membranes. We show that movement of the grafted polymer chins in an oscillating magnetic field will lead to breakup of the concentration polarization boundary layer. The membrane performance was investigated using feed streams consisting of bovine serum albumin (BSA) and dextran in water.

## 2. Material and Methods

### 2.1. Materials

All reagents were ACS grade or higher, unless specified. Methanol, glycerol and acetonitrile were purchased from MilliporeSigma (Billerica, MA, USA). Triethylamine (TEA); 4-dimethylaminopyridine (DMAP); N,N,N′,N″,N″-pentamethyl diethylenetriamine (PMDETA, 99%); sodium phosphate monobasic (99%); sodium phosphate dibasic (99%) copper (I) chloride; copper (II) chloride and copper(I) bromide (CuCl, CuCl_2_ and CuBr 99.999% trace metal basis) were purchased from Sigma-Aldrich (Munich, Germany). α-Bromoisobutyryl bromide (BIB, 98%) was purchased from Alfa-Aesar (Ward Hill, MA, USA). 1,2-epoxy-5-hexene and 2-hydroxyethylmethacrylate (HEMA, >97%, stabilized) was purchased from Acros Organic (98%, Pittsburgh, PA, USA). Bovine serum albumin (BSA, biotechnology grade) and L-(+)-ascorbic acid (AA) were purchased from Amresco (Solon, OH, USA). 2,2′-Bipyridine (Bpy) was purchased from BeanTown Chemical (Hudson, NH, USA). Iron oxide superparamagnetic nanoparticles with a 15-nm core diameter and a 5-nm coating layer functionalized with amine groups were purchased from Ocean Nanotech (San Diego, CA, USA). All water used in this work was obtained from a Thermo Fisher 18 MΩ Barnstead Smart2Pure System (Schwerte, Germany). Commercially available regenerated cellulose membranes with a nominal molecular weight cutoff of 100 kDa were purchased from MilliporeSigma (product codes: PLCHK and PCLMK).

### 2.2. Methods

#### 2.2.1. Modification of RCUF membranes

##### Initiator Immobilization

Membrane discs were washed three times in 25 mL of methanol for 20 min. The membranes were then soaked in deionized (DI) water for 30 min. Acetonitrile was dried over activated molecular sieves before use. The membrane discs were placed in a stirred cell (8050, MilliporeSigma). The cell was filled with glycerol and pressurized to 1.5 bar (21.8 psi). Glycerol was pumped through the membrane pores until 5 mL of permeate were obtained. The membranes were then incubated in glycerol overnight. The membrane surface was patted dry with a Kimwipe and then with a roller wiper in order to remove excess glycerol from the surface of the membrane. Our aim was to fill only the membrane pores with glycerol.

The membranes were placed on a glass plate, and an EPDM gasket with a circular cutout of 35 mm in diameter was centered on the sample (Supplementary Figure S1). A piece of HDPE matching the gasket dimensions was placed on top of the setup, and the assembly was secured with binder clips. A solution of acetonitrile (5 mL) containing 100-mM BIB, 100-mM TEA and 5-mM DMAP was freshly prepared. This reactive solution was added at the top of the membrane and allowed to react for 1 or 5 min at room temperature. After the selected reaction time, the reactive solution was quenched with water and discarded. The membranes were washed in a 1:1 (*v*/*v*) methanol/water mixture 3 times, thus ensuring all glycerol was removed. The modified region was cut out with a 25-mm punch and stored in water overnight.

##### AGET ATRP

After the initiator immobilization, polymer chains were grown from the membrane surface using AGET ATRP. The reaction solution for ATRP consisted of HEMA (2 M), CuCl_2_ (0.02 M) and Bpy (0.05 M) in a 1:9 (*v*/*v*) methanol/water mixture. Ascorbic acid, 0.008 M, was then added. Ascorbic acid is a reducing agent, which is used here to remove dissolved oxygen from the system, and hence, there is no need to deoxygenate the reaction solvent. The color of the solution changed to very dark brown. The membranes were placed in small jars that contained the reactive solution and tightly sealed. The reaction was allowed to occur for the designed time (1 or 4 h) on a shaker table at room temperature.

After the reaction, the polymerization was terminated by adding the membranes to the quenching solution, which consisted of CuBr_2_ (0.22 M) and PMDETA (0.06 M) in a 1:1 (*v*/*v*) methanol/water mixture. The membranes were removed and transferred to a wash solution of 1:1 (*v*/*v*) methanol/water mixture for 2 h. The membranes were again washed and then stored in DI water for 1 h.

##### Monomer Addition

PHEMA-grafted membranes were placed in Schlenck flasks. The flasks were sealed with parafilm, evacuated and backfilled with argon three times. 1,2-epoxy-5-hexane (17.7 mM) and Bpy (28.8 mM) were dissolved in the reaction solvent consisting of a 1:1 (*v*/*v*) methanol/water mixture. The reaction solution was purged with nitrogen for 30 min. Thereafter, copper (I) bromide (5.58 mM) was added to the solution with rapid stirring under argon for 15 min. Finally, 30 mL of the reaction solution was cannulated into each Schlenck flask containing the membrane, and the reaction was allowed to take place at 50 °C for 24 h. After the monomer addition reaction, the reaction was terminated, and the membranes washed with in a 1:1 (*v*/*v*) methanol/water mixture 3 times for 1 h. The membranes were removed from the Schlenck flask and immersed in water overnight.

##### Nanoparticle Attachment

**Figure 1:**
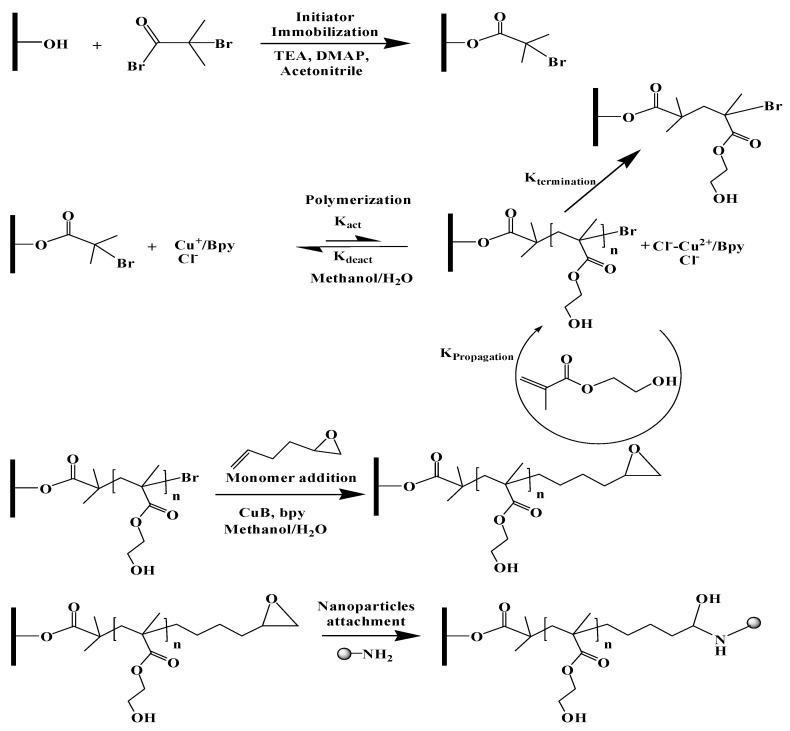
Membrane modification scheme: increasing the time for the initiator immobilization leads to a higher initiator and, thus, grafting density; increasing the polymerization time leads to longer grafted polymer chains [19].

The membranes were rinsed and incubated in 20-mM phosphate buffer at pH 12 for 30 min. The reaction solution was prepared with 15-μL nanoparticle feedstock in 20 mL of 20-mM phosphate buffer at pH 12. The membranes were then added to the reaction solution and incubated for 48 h on a shaker under gentle shaking at room temperature. After 48 h, the membranes were rinsed with water 3 times. The membranes were stored in water prior to use. The overall reaction scheme is summarized in Figure 1.

### 2.3. Membrane Characterization

#### 2.3.1. Attenuated Total Reflectance Fourier-Transform Infrared Spectroscopy (ATR-FTIR)

Prior to analysis, the membranes were dried overnight in a vacuum oven at 40 °C. ATR-FTIR spectroscopy provides qualitative information on the functional groups present to a depth of about 2 µm from the outer membrane surface. Data were obtained using an IR Affinity instrument (Shimadzu, Columbia, MD, USA) with a horizontal ZnSe accessary. ATR-FTIR spectra were averaged over 100 scans covering a range of 1500–4000 cm^−1^.

#### 2.3.2. Atomic Force Microscopy

Prior to analysis, the membranes were dried in a vacuum oven overnight at 40 °C. The surface topography of the modified membranes was measured using a Dimension Icon AFM (Bruker, Santa Barbara, CA, USA). The NanoScope V815R3sr1 program was used to run the AFM, and the NanoScope Analysis program was used to analyze the results. The ScanAsyst mode was used to image the topography of the membranes at room temperature in the air using an etched silicon tip on a nitride lever, which was coated with a 100-nm aluminum layer. The nominal spring constant of the cantilever used was 0.4 N/m and 70 kHz. A standard scan rate of 1 Hz with 512 samples per line was used for imaging the membrane sections. The measured heights of the images were then flattened in order to obtain the final images.

#### 2.3.3. Contact Angle Measurements

Before contact angle measurement, the membranes were dried in a vacuum oven at 40 °C overnight. Contact angles were measured at room temperature with deionized water using a Future Digital Scientific, model OCA15EC (Garden City, NY, USA) contact angle goniometer. The droplet size was 2.0 µL, which was dispensed at a speed of 0.5 µL/s. The contact angle was determined using the circle fitting method, which assumes the droplets are spherical. A small droplet volume was used, allowing us to ignore deviations from spherical droplets due to the effects of gravity. Each value represents the average of 5 replicate measurements.

#### 2.3.4. Size Exclusion Chromatographic (SEC)

Nominal molecular weight cutoff (NMWCO) curves for the modified membranes were determined using an Agilent Technologies Model 1050 HPLC System using an Agilent Technologies 1047A Refractive Index Detector (Palo Alto, CA, USA). The SEC column was purchased from Showa Denko (model no. SB-806M HQ, Tokyo, Japan). The main column was preceded by a Showa Denko SB-G guard column. The column temperature was maintained at 40 ± 1 °C. The feed solution consisted of various molecular weight dextrans (6 kDa, 10 kDa, 50 kDa, 70 kDa, 100 kDa, 250 kDa and 500 kDa at an individual concentration of 1 mg/mL). The dextran standards were dissolved in 0.05-mol/L reagent-grade NaH_2_PO_4_ (Sigma-Aldrich, St. Louis, MO, USA). The same buffer was used as the mobile phase for the SEC analysis. The rejection curve corresponding to the rejection (*R*) of each molecular weight was calculated according to Equation (1):

(1) $$R=1-\;\frac{C_{p}}{C_{f}}\;\times \;100\%$$

where *C_p_* and *C_f_* and correspond to the permeate and feed concentrations. The corresponding NMWCO was subsequently taken as the molecular weight that gave a 90% rejection.

### 2.4. Membrane Performance

Testing was conducted at room temperature in dead-end filtration mode using an Amicon 8010 stirred cell (MilliporeSigma). Pressurized nitrogen was used to drive the feed solution through the membrane. All tests were conducted without stirring. Prior to flux measurements, the membranes were soaked in methanol for 15 min, then DI water for a further 15 min. The membranes were tested in the presence and absence of an oscillating magnetic field.

The oscillating magnetic field was generated using a custom-built system. The stirred cell was placed between two solenoids. A computer-controlled programmable logic controller (PLC, Click Koya Automation Direct, Cumming, GA, USA) controlled the two solenoids by alternatively activating two solid-state relays. Thus, the frequency of the alternating magnetic field could be set. The solenoids were powered by an Agilent Technologies (Santa Clara, CA, USA) 20 V/25 A power supply. The solenoids were placed on opposite sides of the filtration cell so that the magnetic field was parallel to the barrier layer of the membrane. The oscillation frequency of the magnetic field was set at 20 Hz, and the current was set at 2 A. Our previous studies indicate that these conditions maximize the movement of the grafted polymer chains for the superparamagnetic nanoparticles used here [12,13]. Insulated foam was placed between the solenoids and the stirred cell in order to prevent heat transfer to the stirred cell from the solenoid. The permeate flux was determined by weighing the permeate using a Mettler Toledo PL 602~S (Columbus, OH, USA) balance that was connected to a computer. The experimental set up is shown in Supplementary Figure S2.

Water flux data were collected when a stable flux was obtained after the first few minutes of operation. A feed pressure of 0.4 bar (5.8 psi) was used. Rejection of dextran and BSA was investigated. For dextran rejection, 1 mg mL^−1^ of dextran 70 (average molecular weight 70 kDa) solution was prepared as the stock solution using DI water. Total organic carbon analysis (TOC) was used to determine the dextran concentration in the feed and permeate. The % rejection (R) of dextran was calculated according to Equation (1). A feed pressure of 0.4 bar (5.8 psi) was used.

For BSA rejection measurements, the feed solution was prepared by adding 1-mg mL^−1^ BSA to DI water. BSA solutions at different pH were prepared according to the procedure reported in the literature [20]. The membranes were soaked in DI water for 30 min and loaded into the stirred cell. The feed solution was loaded into the feed reservoir. A pressure of 0.4 bar (5.8 psi) was used. The relative concentration of the feed and permeate was measured by comparing the UV absorbance at 280 nm. The BSA rejection was calculated as follows:

(2) $$R=\left(1-\;\frac{Absorbance\;of\;permeate\;solution\;at\;280\;\mathrm{nm}}{Absorbance\;of\;feed\;solution\;at\;280\;\mathrm{nm}}\right)\;\times \;100$$

The permeate fluxes for dextran and BSA feed solutions initially decreased at the start of the filtration. After about 5 min of operation, a pseudo-steady state flux was obtained for all the membranes tested, where the flux did not decrease significantly during operation. This flux was recorded.

## 3. Results and Discussion

### 3.1. Characterization

#### 3.1.1. FTIR Analysis

**Figure 2:**
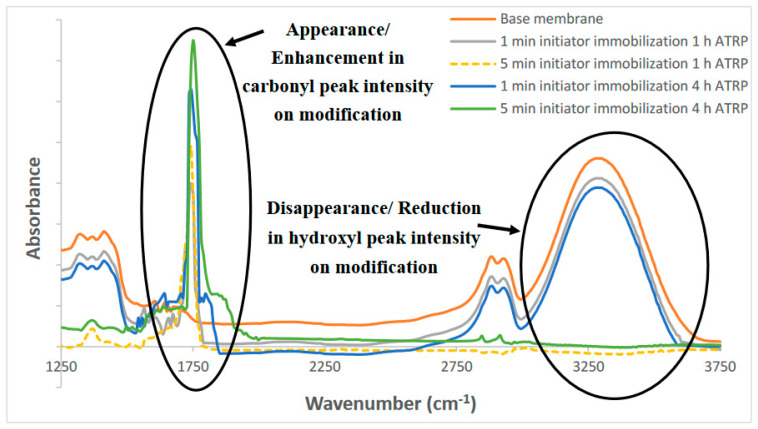
Attenuated total reflectance Fourier-transform infrared (ATR-FTIR) spectra for the base and modified membranes. ATRP: atom transfer radical polymerization.

FTIR spectra for the base and modified membranes are given in Figure 2. The peak at 3340 cm^−1^ represents hydroxyl groups present in the base RCUF membranes. The intensity of the peak decreases upon modification. PHEMA would also introduce hydroxyl functionalities. However, the hydroxyl moieties from the polymer chains may not be exposed properly on the surface, resulting in the overall reduction in intensity of the –OH peak on modification. The greater the degree of modification, the greater the decrease in the peak as the amount of PHEMA grafted from the membrane surface increases. This is due to the presence of a thicker grafted nanolayer. For the modified membranes, a peak appears at ~1735 cm^−1^, which may be assigned to the carbonyl group present in the PHEMA nanostructure that is grafted from the membrane surface. The intensity of this peak increases as the amount of PHEMA grafted from the membrane surface increases [13]. Amide I and II peaks resulting from the attachment of the nanoparticles to the PHEMA chains are not observed, probably due to the low density of the amide groups compared to the carbonyl groups.

#### 3.1.2. Contact Angle

**Figure 3:**
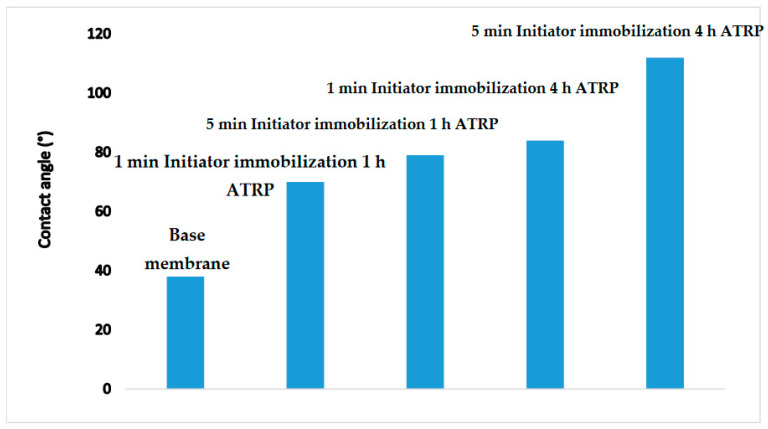
Water contact angles (°) for the base and modified membranes (error within ±5°).

Water contact angles for the base and modified membranes are given in Figure 3. As can be seen, the base membrane is very hydrophilic. As the amount of PHEMA grafted from the membrane surface increases, the contact angle increases. Increasing the chain density (initiator immobilization time) and chain length (polymerization time) leads to an increase in grafted PHEMA. Further, the membrane surface is now covered with nanoparticles. The increase in the contact angle reflects the increased roughening of the membrane surface due to the presence of nanoparticles, as well as the contact angle of the amine coating on the nanoparticles and PHEMA. The hydrophilicity of the –OH groups is greater compared to that of the –NH_2_ groups. Moreover, the surface iof the modified membrane may also be exposed with the organophilic polymer chains.

#### 3.1.3. AFM Analysis

**Figure 4:**
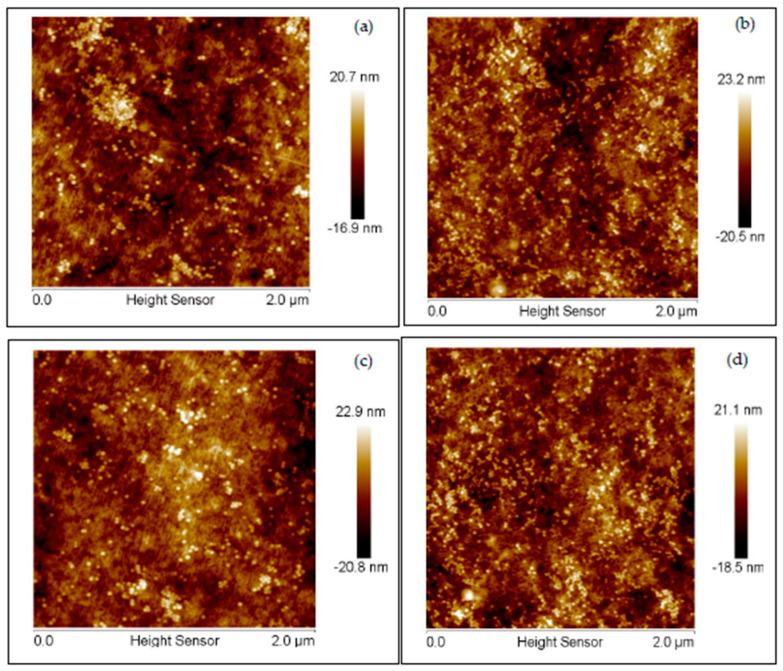
AFM images for modified membranes: (**a**) 1-min initiator immobilization and 1-h ATRP, (**b**) 5-min initiator immobilization and 1-h ATRP, (**c**) 1-min initiator immobilization and 4-h ATRP and (**d**) 5-min initiator immobilization and 4-h ATRP.

AFM images are given in Figure 4. These images indicate qualitatively that five min of initiator immobilization leads to a greater number of nanoparticles being attached to the membrane surface compared to a one-min immobilization time. A longer initiator immobilization time will lead to an increase in the number of initiator molecules that are attached to the membrane surface and, hence, the number of grafted polymer chains (higher grafting density). As nanoparticles are only attached to the end of the polymer chains, a greater number of polymer chains is likely to lead to a greater number of attached nanoparticles, as indicated in Figure 4.

On the other hand, the polymerization time appears to have little effect on the number of attached nanoparticles. This is expected, as an increased polymerization time will lead to longer grafted polymer chains but not an increase in the grafting density. As we have noted earlier [13], this highlights our ability to independently vary the polymer chain density and chain length (cf. Figure 1). The images indicate that the nanoparticles are around 25 nm, as stated by the manufacturer. An AFM image of the base RCUF membrane is given in Supplementary Figure S3.

#### 3.1.4. SEC for Barrier Pore Size Determination

**Figure 5:**
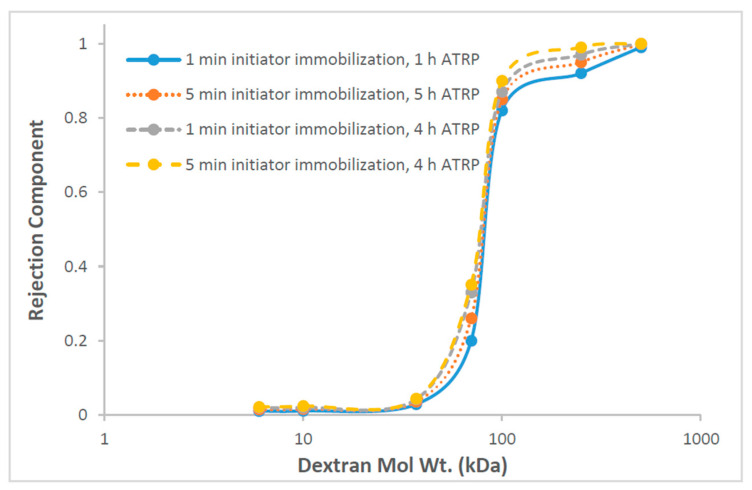
Dextran rejection as function of molecular weight for membranes modified under different conditions.

Dextran rejection curves for the modified membranes are given in Figure 5. These experiments were conducted in the absence of an oscillating magnetic field. For the base 100-kDa membrane, the NMWCO for 90% rejection is 115 kDa. Table 1 gives the NMWCO for all the membranes. Our results indicate that the barrier pore size of the membranes was not significantly modified, since the rejection curves for the modified membranes are similar. Further, the NMWCO for the modified membranes is similar to the base membranes.

### 3.2. Membrane Separation Performance

In a magnetic field, the superparamagnetic particles will experience a force and a torque [12,18]. The force will lead to transnational movement, while the torque will result in rotation of the particles. It is the translational movement of the tethered nanoparticles that is the focus here. In an oscillating magnetic field, the back and forth movement of the tethered superparamagnetic nanoparticles will lead to mixing. Movement ceases when the field is removed. The effect is completely reversible.

Table 2 gives the steady-state DI water flux. The flux reached a steady-state value a few minutes after the start of each run. The water flux of the unmodified membrane was 250 L m^−2^ h^−1^. Table 3 gives the pseudo-steady-state permeate flux during the dead-end filtration of BSA. Table 4 gives the corresponding rejection of BSA. Table 5 and Table 6 give the pseudo-steady-state flux and rejection for the dead-end filtration of dextran 70.

The results indicate that increasing the chain density (via the initiator immobilization time) and chain length (via the polymerization time) will lead to a decrease in the permeate flux and an increase in the apparent rejection coefficient. While using glycerol as a pore-filling solvent suppresses initiator immobilization inside the membrane pore surface, it is likely that the initiator molecules will attach to the membrane pore mouth. Grafting at the pore mouth will lead to a constriction of the pore, resulting in a lower permeate flux. As the grafted chain density and length increase, this effect will increase. The slight decrease in the NMWCO of the modified membranes (Table 1) supports this observation. The decrease in the permeate flux and increase in the apparent rejection coefficient are much more significant for BSA compared to dextran 70. BSA is a globular protein with a molecular weight of 67 kDa, while dextran 70 is made up of linear dextran molecules with an average molecular weight of 70 kDa. It is expected, therefore, that the apparent rejection coeffect for a globular molecule will be higher than for linear molecules of the same molecular weight.

Table 2 indicates that, in the presence of an oscillating magnetic field, the DI water flux decreases for all four modified membranes. The same trend is observed for the permeate flux during BSA and dextran 70 filtration (Table 3 and Table 5, respectively). In our earlier work, we grafted the same polymer chains from the surface of PET track-etched membranes [17]. These membranes had a nominal pore size of 0.4 μm. No pore-filling solvent was used, as the aim was to graft magnetically responsive polymer chains from the inside pore surface.

In an oscillating magnetic field, a decrease in permeability was observed analogous to the results obtained here. In the presence of an oscillating magnetic field, it is likely the polymer chains will, on average, adopt a more stretched conformation, which will bridge across the pore mouth more effectively compared to their relaxed conformation in the absence of a magnetic field. This could lead to an increase in the resistance to the permeate flow and, hence, a decrease in the permeate flux at the same feed pressure.

As indicated by Table 4 and Table 6 in the presence of an oscillating magnetic field, an increase in the rejection coefficient for BSA and dextran is observed. As noted above, this could be due to the polymer chains adopting a more stretched conformation that leads to a greater bridging across the pore mouth. In addition, in our earlier work, we have shown that, for nanofiltration membranes, movement of the magnetically responsive polymer chains leads to a breakup of the concentration polarization boundary layer. This, in turn, leads to an increase in the apparent rejection coefficient for partially rejected solutes [16,17,18]. It is possible that both effects are responsible for the increase in the rejection of dextran and BSA.

Breakup of the concentration polarization boundary layer at the membrane surface could suppress fouling and increase the volume of feed that can be treated prior to membrane cleaning, as we have shown for nanofiltration membranes [15,16]. This could be beneficial during protein concentration. It could also enable the efficient ultrafiltration of higher concentration protein solutions where fouling by rejected feed components limits the membrane performance. Our previous work on developing magnetically responsive nanofiltration membranes for wastewater treatment [16] suggests the power costs will be low, but the cost associated with establishing an oscillating magnetic field will impact the economic viability of the process. The results indicate the importance not only of carefully controlled polymer grafting but, also, control of the location of the grafted polymer chains. Relatively monodisperse polymer chains grafted from the outer membrane surface and not the pore surface are essential.

## 4. Conclusions

For the first time, magnetically responsive polymer chains have been grafted from the surface of ultrafiltration membranes where a superparamagnetic nanoparticle is attached to the chain end. By suppressing the initiator immobilization within the membrane pores, plugging of the membrane pores is suppressed. Dextran rejection curves indicate that the NMWCO of the membranes is not significantly affected by the modification. By choosing a frequency that maximizes the movement of the superparamagnetic nanoparticles in an oscillating magnetic field, the particles induce movement of the grafted polymer chains. This movement has been shown to lead to a decrease in the permeate flux between 22–41% for BSA and 15–48% for dextran for the modification conditions investigated. A concurrent increase in the rejection coefficient of 5.5 and 8.2% for BSA and 4.0 and 16.0% for dextran was observed. Consequently, the membrane performance may be modulated by applying an external magnetic field.

## Figures and Tables

**Table 1:** Nominal molecular weight cutoff (NMWCO) of modified membranes for 90% dextran rejection. ATRP: atom transfer radical polymerization.

Immobilization Time (min)	ATRP Time (h)	NMWCO (kDa)
1	1	115
5	1	112
1	4	110
5	4	100

**Table 2:** Steady state deionized (DI) water flux (L m^−2^ h^−1^) during dead-end filtration.

Magnetic Field (Hz)	Initiator Immobilization Time (min)	Flux	
		Polymerization Time 1 h	Polymerization Time 4 h
0	1	217	75
20	1	177	63
0	5	150	69
20	5	128	37

**Table 3:** Pseudo-steady-state flux (L m^−2^ h^−1^) during 1 mg/mL of bovine serum albumin (BSA) filtration.

Magnetic Field (Hz)	Initiator Immobilization Time (min)	Flux	
		Polymerization Time 1 h	Polymerization Time 4 h
0	1	170	32
20	1	132	23
0	5	123	29
20	5	86	17

**Table 4:** BSA rejection (%) by modified membranes.

Magnetic Field (Hz)	Initiator Immobilization Time (min)	Rejection	
		Polymerization Time 1 h	Polymerization Time 4 h
0	1	62.1	75.6
20	1	67.6	80.1
0	5	66.1	83.3
20	5	74.3	90.2

**Table 5:** Pseudo-steady-state flux (L m^−2^ h^−1^) during 1 mg/mL of dextran (70 kDa) filtration.

Magnetic Field (Hz)	Initiator Immobilization Time (min)	Flux	
		Polymerization Time 1 h	Polymerization Time 4 h
0	1	206	70
20	1	170	57
0	5	141	60
20	5	119	31

**Table 6:** Dextran rejection (%) by modified membranes.

Magnetic Field (Hz)	Initiator Immobilization Time (min)	Rejection	
		Polymerization Time 1 h	Polymerization Time 4 h
0	1	4.2	15.2
20	1	8.1	24.8
0	5	8.2	22.8
20	5	13.7	38.9

## Supplementary Materials

The following are available online at https://www.mdpi.com/2077-0375/10/9/219/s1, Figure S1: Schematic representation of the initiator immobilization protocol, Figure S2: Experimental set up for testing modified membranes, Figure S3: AFM images for 100 kD base RCUF membrane.
