# Supplementary material for: Tailoring and Remotely Switching Performance of Ultrafiltration Membranes by Magnetically Responsive Polymer Chains
Source: Membranes (Basel). 2020 Sep 1;10(9):219. doi: 10.3390/membranes10090219 (PMC7558725; doi:10.3390/membranes10090219)
Supplement: Supplementary file 1 [file membranes-10-00219-s001.pdf]

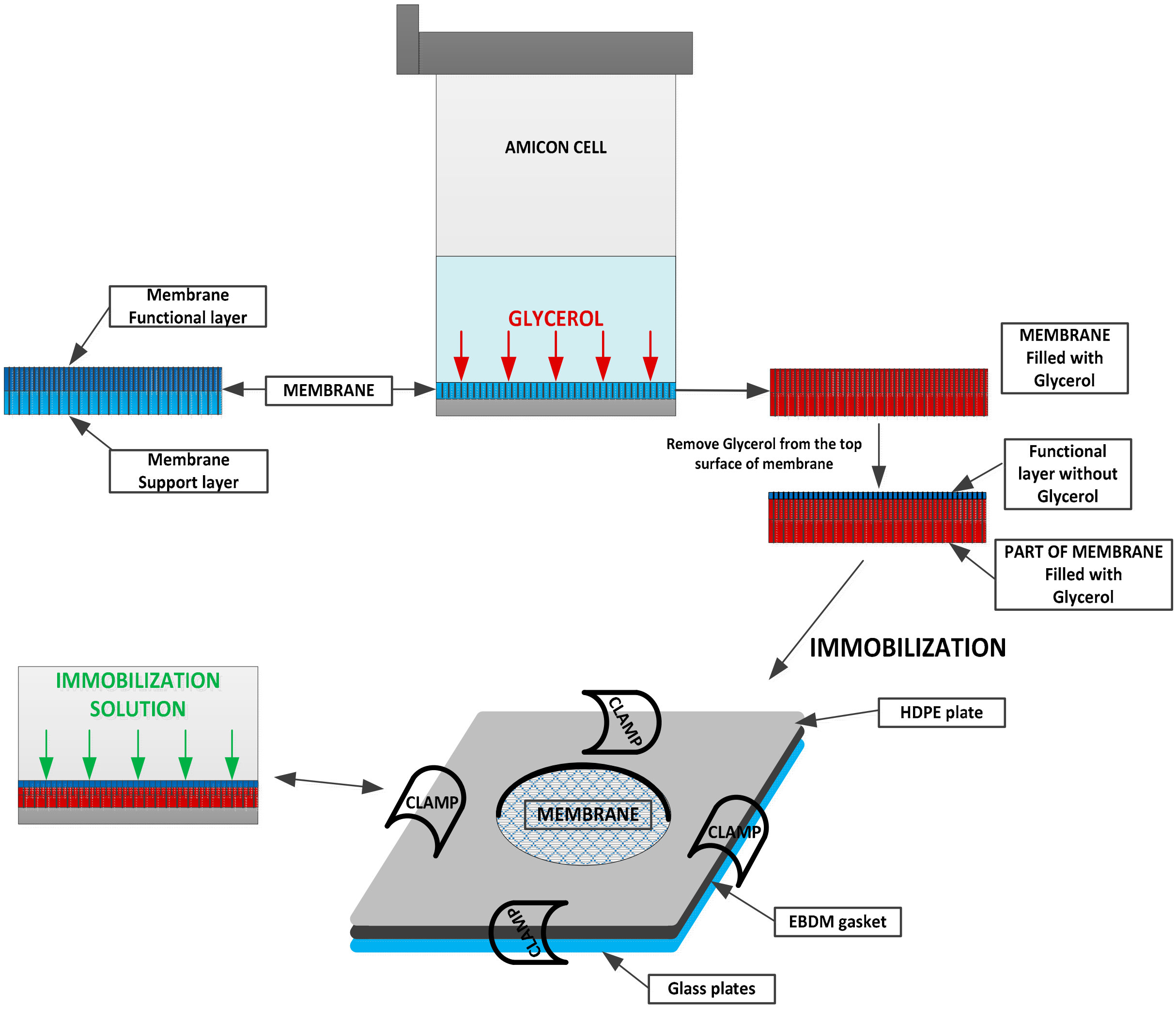

Supplementary Fig. 1: Schematic representation of the initiator immobilization protocol.

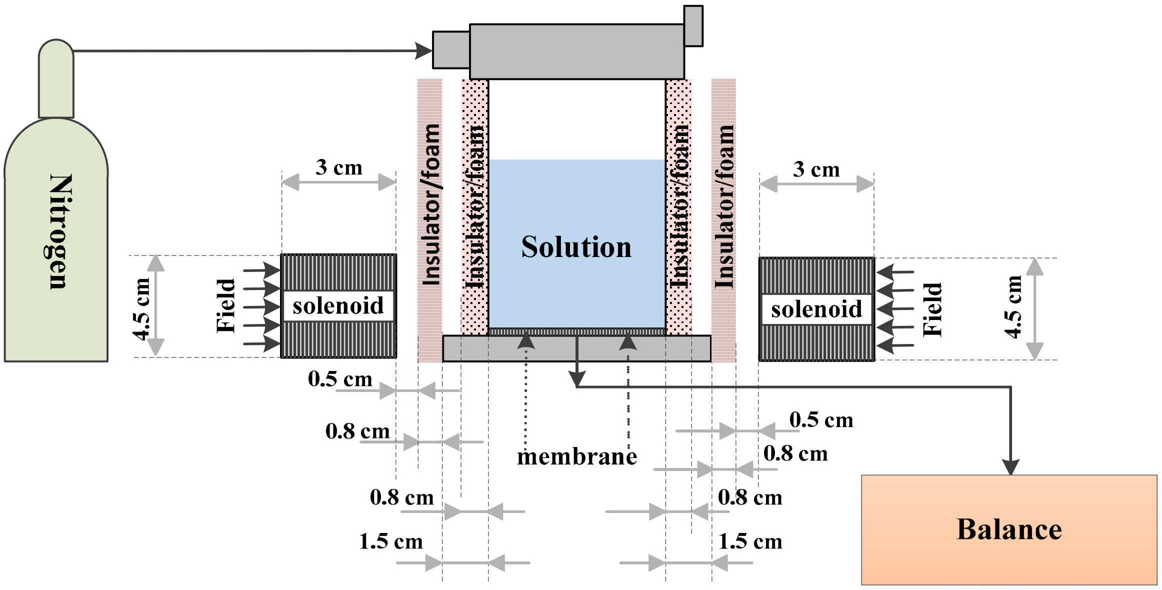

Supplementary Fig. 2: Experimental set up for testing modified membranes.

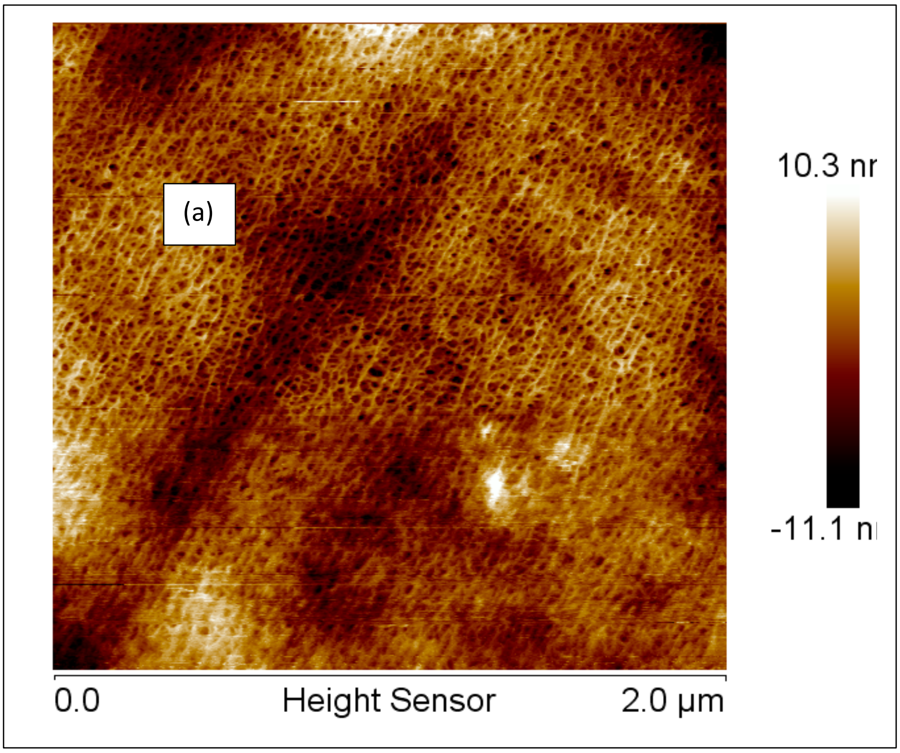

Supplementary Fig. 3: AFM images for 100 kD base RCUF membrane.
